# Osteomyelitis: A Case in Practice

**Published:** 1904-02

**Authors:** H. B. Hickman

**Affiliations:** Philadelphia


### OSTEOMYELITIS: A CASE IN PRACTICE.[1]

BY DR. H. B. HICKMAN, PHILADELPHIA.

THE case I wish to bring before you is that of a man, aged forty-three years, weighing one hundred and sixty pounds, five feet six inches tall, with no apparent evidence of any specific disease, although his mother died of some form of tuberculosis.

Up to within six months of the time when this second bicuspid started to give trouble, he was in fairly good health, being manager of a large business and with a superabundance of energy, although he had not taken a vacation for eighteen years.

His wife had been in bed with an incurable disease for over a year; he was needed abroad to attend to very important business, but his wife would not consent to his going away even for a short stay; and since his wife had been sick he had been forced to give up his only recreation, music. The strain at home and office, with no rest, began to manifest itself by his being very nervous, having lost appetite, being easily fatigued and anæmic.

I was able to watch the change in the condition of this man because he was a very intimate friend, and also because his teeth gave him a great deal of annoyance during this period; but he would not listen to advice as to rest, tonics, etc.

This brings us to the time he called at the office with the right lower second bicuspid very sore, aching, and slightly loose. I diagnosed pericementitis caused by a putrescent pulp. After chilling the tooth with chloride of ethyl spray, which relieved the pain, I opened through a small amalgam filling and found the pulp-chamber and canal filled with gutta-percha; then the patient remembered that it had been treated and filled ten or fifteen years before. After removing the root-canal filling I was able to pass a broach through the apical foramen, which opening gave only momentary relief.

I prescribed a laxative and anodyne, with the usual treatment for pericementitis, feeling sure he would be better the next morning.

On the day following he returned to the office. The tooth had ached continuously and he wished it extracted, which was very

---

[1] Read before the Academy of Stomatology, Philadelphia, October 27, 1903.

nicely done, under nitrous oxide gas, by a specialist, who cleansed the mouth with an antiseptic solution both before and after extracting. I examined the root and found it slightly exostosed. The socket was packed with phenol sodique and cotton.

The pain returned before he reached home, and his physician, who had been summoned, could not relieve him. His physician wished him to go to bed there and then, but he suffered so much more when lying down that he slept in a reclining-chair.

I saw him at his home the following day at noon, and found that a saturated solution of carbolic acid on cotton gave him relief for eight hours, when the pain returned with renewed force.

This condition continued for ten days, the socket being packed every eight hours with carbolic acid, at the same time using fifty per cent. phenol sodique as a mouth-wash.

Sulphate of zinc was tried in the socket with only partial relief.

By this time the patient was in bed with a temperature varying from 102° to 104°. The legs and body were covered with pustules resembling chicken-pox, which disease the physician led the family to believe he had.

At this time there was some swelling or puffiness of the gum tissue around the socket, which had not filled up very much. The temperature was about 103° to 104°. There were rigors alternating with flashes of heat, followed by copious sweats, which had rather a disagreeable odor. He had an exceedingly irritating cough, especially when he talked or became the least excited.

At the end of six weeks, a mere shadow of his former self, he died, having been delirious the greater part of the last five weeks.

According to one physician it was called general nervous collapse, but others thought it was septic meningitis that caused his death, superinduced by the osteomyelitis and the very low state of his health.

